# Evaluating the causality of novel sequence variants in the prion protein gene by example

**DOI:** 10.1016/j.neurobiolaging.2018.05.011

**Published:** 2018-11

**Authors:** Tze How Mok, Carolin Koriath, Zane Jaunmuktane, Tracy Campbell, Susan Joiner, Jonathan D.F. Wadsworth, Laszlo L.P. Hosszu, Sebastian Brandner, Ambereen Parvez, Thomas Clement Truelsen, Eva Løbner Lund, Romi Saha, John Collinge, Simon Mead

**Affiliations:** a MRC Prion Unit, UCL Institute of Prion Diseases, London, UK; b National Prion Clinic, National Hospital for Neurology and Neurosurgery, UCLH NHS Foundation Trust, London, UK; c Department of Neurodegenerative Diseases, UCL Institute of Neurology, London, UK; d Department of Molecular Neuroscience, UCL Institute of Neurology, London, UK; e Division of Neuropathology, National Hospital for Neurology and Neurosurgery, UCLH NHS Foundation Trust, London, UK; f Department of Neurology, University of Copenhagen, Rigshospitalet, Copenhagen, Denmark; g Department of Pathology, Rigshospitalet, Copenhagen, Denmark; h Hurstwood Park Neurological Centre, Sussex, UK

**Keywords:** Inherited prion disease, Creutzfeldt-Jakob disease, Prion protein, Rare gene variant, Novel gene variant interpretation

## Abstract

The estimation of pathogenicity and penetrance of novel prion protein gene (*PRNP*) variants presents significant challenges, particularly in the absence of family history, which precludes the application of Mendelian segregation. Moreover, the ambiguities of prion disease pathophysiology renders conventional in silico predictions inconclusive. Here, we describe 2 patients with rapid cognitive decline progressing to akinetic mutism and death within 10 weeks of symptom onset, both of whom possessed the novel T201S variant in *PRNP*. Clinically, both satisfied diagnostic criteria for probable sporadic Creutzfeldt-Jakob disease and in one, the diagnosis was confirmed by neuropathology. While computational analyses predicted that T201S was possibly deleterious, molecular strain typing, prion protein structural considerations, and calculations leveraging large-scale population data (*gnomAD*) indicate that T201S is at best either of low penetrance or nonpathogenic. Thus, we illustrate the utility of harnessing multiple lines of prion disease–specific evidence in the evaluation of the T201S variant, which may be similarly applied to assess other novel variants in *PRNP*.

## 1 Introduction

Prion diseases are transmissible, fatal neurodegenerative conditions affecting humans and animals (Collinge, 2001). The infectious agent is composed of assemblies of abnormally folded host-encoded prion protein (PrP), some of which acquire protease resistance, designated as PrP^Sc^ (Prusiner, 1998). Human prion diseases most commonly occur sporadically but can be acquired through dietary exposure or iatrogenesis. Inherited prion diseases (IPDs) comprise 10%–15% of the total annual incidence and are associated with coding mutations in the prion protein gene (*PRNP*) (Mead, 2006). Clinical phenotypes of IPD are highly heterogeneous and include rapidly progressive forms of dementia and/or ataxia (indistinguishable from sporadic Creutzfeldt-Jakob disease [CJD]), fatal familial insomnia and more slowly progressive syndromes such as Gerstmann-Straussler-Scheinker disease and PrP systemic amyloidosis (Mead and Reilly, 2015, Mead et al., 2013).

A great deal is now known about normal variation of *PRNP* in different populations (Beck et al., 2010, Minikel et al., 2016). Situated on one end of the spectrum are commonly occurring benign polymorphisms, some of which can modify prion disease susceptibility and clinical phenotype, while on the other lies well-defined highly penetrant variants such as the P102L, E200K, D178N, and large octapeptide repeat insertions. Then there are partially penetrant variants such as V210I found both in control and patient populations that are associated with increased risk but not inevitable disease (Minikel et al., 2016). The advent of low-cost, high-throughput genomic sequencing technologies has led to large-scale population genomic databases that can be used to estimate penetrance. Such an approach used recently led to reclassifying several *PRNP* sequence variants, previously reported to be pathogenic in the literature, as likely to be either low risk or even benign (Minikel et al., 2016).

The most challenging ones to classify are the extremely rare variants found in only a few patients and controls. Causal analyses of these rare *PRNP* variants seen in CJD, particularly in the absence of family history, have historically been biased toward overcalling of pathogenicity (Minikel et al., 2016). Erroneous assignation of pathogenicity and penetrance to a benign variant may not only lead to unnecessary psychological distress but could also misdirect genetic counseling for the patients' relatives. At the research level, analysis of sets of variants classified as accurately as possible by pathogenicity may help uncover fundamental mechanisms of prion disease. Here, we illustrate our practice in estimation of the causality of the novel *PRNP* variant T201S. We used multiple lines of evidence and address the challenges faced with interpretation of rare gene variants that may be applicable to other *PRNP* variants and those in genes related to other neurodegenerative diseases.

## 2 Methods

### 2.1 Neuropathology

Formalin-fixed and formic acid pretreated paraffin-embedded postmortem brain tissue samples were available from case 1. Tissue sections from the neocortex, hippocampus, deep gray nuclei, brain stem, and cerebellum were routinely stained with hematoxylin and eosin and PrP immunohistochemistry (anti-PrP antibodies ICSM35, D-Gen Ltd, London, UK, 1:1000 and KG9, University of Edinburgh, 1:500) with Ventana (Roche) automated staining instruments following the manufacturer's guidelines, using biotinylated secondary antibodies and a horseradish peroxidase–conjugated streptavidin complex and diaminobenzidine as a chromogen.

### 2.2 Immunoblotting and molecular genetic and strain typing

All procedures were carried out in a microbiological containment level III facility with strict adherence to safety protocols. Frozen brain (gray matter from frontal cortex) of case 1 was prepared as a 10% (w/v) homogenate in Dulbecco's sterile phosphate-buffered saline lacking Ca^2+^ and Mg^2+^ ions using a tissue grinder as described previously (Wadsworth et al., 2008). The brain homogenate was analyzed with and without proteinase K digestion (50 μg/mL final protease concentration, 1 hour, 37 °C) by immunoblotting with anti-PrP monoclonal antibody 3F4 using high-sensitivity enhanced chemiluminescence as described previously (Wadsworth et al., 2001, Wadsworth et al., 2008). Molecular strain typing of PrP^Sc^ was performed by comparison to reference cases of sporadic CJD (sCJD) and IPD of known PrP^Sc^ type (Hill et al., 2006, Hill et al., 2003). For quantitation and analysis of PrP^Sc^ glycoform ratios, blots were developed in chemifluorescent substrate (AttoPhos; Promega) and visualized on a Storm 840 phosphorimager (Molecular Dynamics). Quantitation of PrP^Sc^ glycoforms was performed using ImageQuaNT software (Molecular Dynamics) (Hill et al., 2003, Hill et al., 2006, Wadsworth et al., 2008). Gene analysis was done as previously described (Wadsworth et al., 2008).

## 3 Results

### 3.1 Case 1

A 63-year-old right-handed Danish Caucasian lady, with no previous medical illnesses or family history of neurodegenerative diseases, was admitted urgently to her local stroke unit with a 5-day history of abrupt onset fluent dysphasia in October 2009. Detailed speech examination revealed preserved fluency and comprehension but markedly impaired repetition, reminiscent of conduction aphasia. Computed tomography of her brain was unremarkable, and she was subsequently discharged with secondary prevention measures for stroke, after 3 days. Nine days following hospital discharge, she returned with sudden onset right-sided paresthesia, and thereafter, her clinical complex evolved rapidly through a sequence of dysarthria, nonfluent speech, dyslexia, dysgraphia, motor and verbal perseveration, startle, myoclonus, akinetic mutism, and finally death over the period of 10 weeks.

The patient has 2 older sisters, both of whom are alive and well in their 70s at the present time. Her father died of cancer at the age of 80 years, while her mother lived until the age of 90 years; neither parent had neurological or cognitive symptoms in life. The patient's father had a sister who died in “old age” of an unknown cause; her mother had 2 other siblings who died of cancer at 63 and 73 years of age, respectively.

Magnetic resonance imaging (MRI) of her brain revealed restricted diffusion in her caudate heads, anterior putamina, and predominantly left-sided cortical ribboning. Her electroencephalogram (EEG) showed left frontotemporal slowing of 1–2 Hz, with occasional sharp waves over the left hemisphere. Her cerebrospinal fluid had 3 white cells and 308 red cells but normal protein and glucose levels; protein 14.3.3 was positive, and neuron-specific enolase (NSE) was raised 101 ng/ml (<35 ng/mL); real-time quaking-induced conversion assay was not performed.

### 3.2 Case 2

A 76-year-old right-handed British Caucasian woman, with no family history of neurodegenerative diseases, developed abrupt onset bilateral upper limb postural and action myoclonus. In the following week, she exhibited unusual sitting postures (axial apraxia), and her gait assumed a narrow-based shuffling character (gait apraxia). She then developed a rapidly progressive nonfluent dysphasia that rendered her effectively mute within 3 weeks. In tandem with that, she became socially withdrawn, abulic, and completely indifferent to her surroundings. Subsequently, she developed visual hallucinations, exaggerated startle, severe myoclonus, incontinence, and akinetic mutism. She died 8 weeks after symptom onset; a postmortem examination was not carried out.

The patient was the only child. Her father died of bone cancer at the age of 73 years, while her mother died of lung cancer at the age of 57 years.

Her MRI brain showed asymmetrical cortical ribboning with a left-sided emphasis and bilateral anterior basal ganglia diffusion restriction, while her EEG showed generalized periodic complexes. cerebrospinal fluid cell count and routine biochemistry were normal, but no sample was analyzed for protein 14.3.3, S100B, or real-time quaking-induced conversion assay.

### 3.3 PRNP analysis

Sequencing of the open reading frame of the *PRNP* in both patients demonstrated a threonine to serine missense substitution at codon 201 (T201S); the underlying nucleotide change was c.602C>G (CCDS 13080.1) in both cases. Their codon 129 genotypes were both methionine homozygous (MM).

### 3.4 Neuropathology

**Fig. 1:**
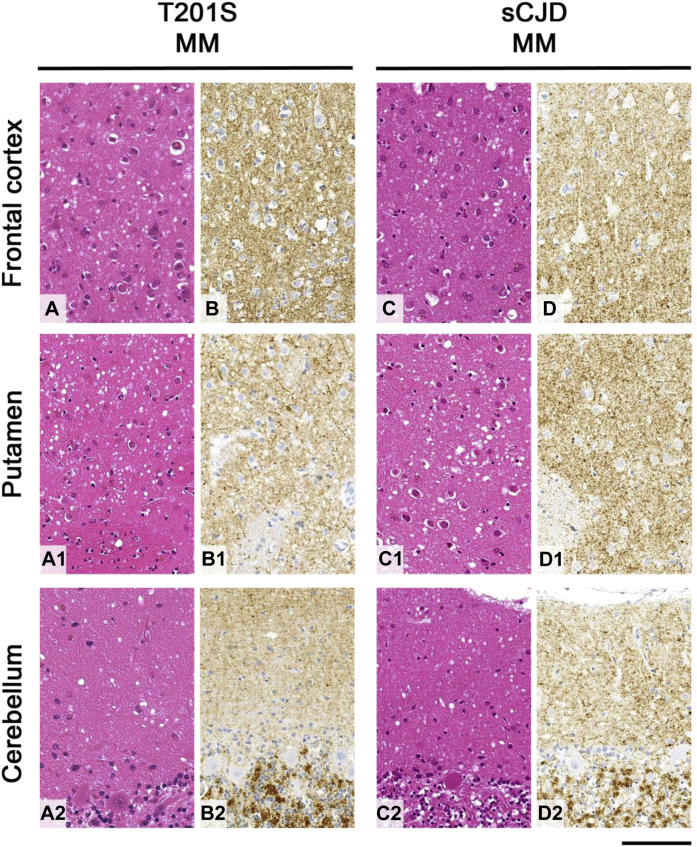
Comparison of prion pathology between T201S patient (case 1) and classical sCJD case, both with *PRNP* codon 129MM genotype. Prion pathology in T201S patient (A–A2 and B–B2) is similar to that seen in *PRNP* 129MM sCJD case (C–C2 and D–D2): Hematoxylin and eosin–stained sections from the frontal cortex (A and C), putamen (A1 and C1), and cerebellar cortex (A2 and C2) show widespread microvacuolar degeneration in the neuropil. The same regions immunostained for abnormal PrP with KG9 antibody (B–B2) and ICSM35 antibody (D–D2) show diffuse synaptic (punctate or granular) labeling (B and D, frontal cortex), (B1 and D1, putamen), and (B2 and D2, cerebellar cortex). Scale bar: 100 μm. Abbreviations: MM, methionine homozygous; PrP, prion protein; sCJD, sporadic Creutzfeldt-Jakob disease.

Routine hematoxylin and eosin–stained sections revealed widespread microvacuolar degeneration in the neocortex, deep gray nuclei and to a lesser extent in the molecular layer of the cerebellar cortex. Immunostaining for abnormal PrP showed diffuse synaptic (punctate or granular) labeling throughout gray matter regions but no kuru or multicentric plaques or other plaque-like deposits (Fig. 1). In the white matter, there were no filamentous deposits, which have been reported in a proportion of IPD cases (Reiniger et al., 2013). The histological appearances were indistinguishable from sCJD patients with *PRNP* 129MM genotype and type 2 molecular prion strain [London Classification (Hill et al., 2003) corresponding to type 1 of the Parchi classification (Parchi et al., 2009)].

### 3.5 Molecular strain typing

**Fig. 2:**
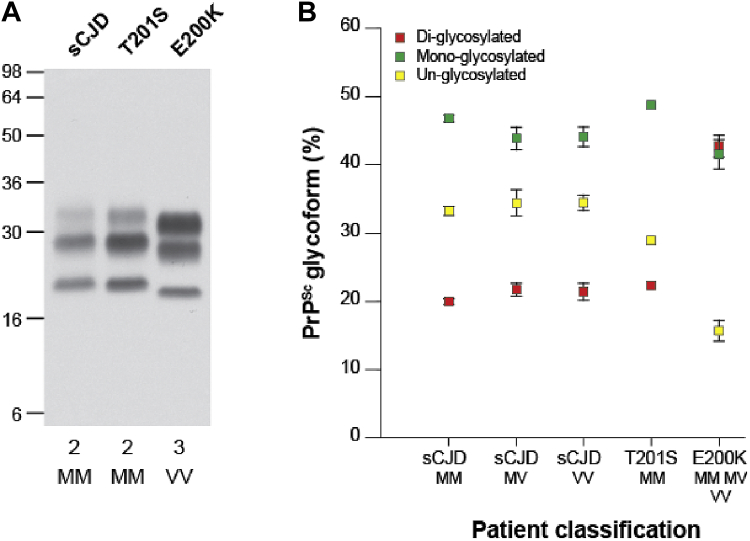
PrP^Sc^ typing in T201S patient brain. (A) Immunoblot of proteinase K–digested 10% (w/v) brain homogenates (frontal cortex) from T201S case 1 and reference cases of sCJD or IPD E200K using anti-PrP monoclonal antibody 3F4 and high-sensitivity enhanced chemiluminescence. The provenance of the brain sample is designated above each lane and the PrP^Sc^ type (London classification [Hill et al., 2006, Hill et al., 2003]) and *PRNP* codon 129 genotype of the patient (M, methionine, V, valine) are shown below. (B) Ratios of the 3 principal protease-resistant PrP glycoforms seen in PrP^Sc^ from T201S case 1 in comparison to PrP^Sc^ from patients with classical CJD or IPD E200K. Data points for the reference cases represent the mean relative proportions of diglycosylated, monoglycosylated, and unglycosylated PrP as percentage ± standard error of measurement. In some cases the error bars were smaller than the symbols used. The number of reference cases analyzed were sCJD 129MM with type 2 PrP^Sc^ (n = 37), sCJD 129MV with type 2 PrP^Sc^ (n = 8), sCJD 129VV with type 2 PrP^Sc^ (n = 9), and IPD E200K (n = 6; three 129MM with type 1 PrP^Sc^ fragment size, two 129MV with type 2 PrP^Sc^ fragment size and 1 129VV with type 3 PrP^Sc^ fragment size). Abbreviations: CJD, Creutzfeldt-Jakob disease; PrP, prion protein; PK, proteinase K; sCJD, sporadic CJD.

Immunoblot analyses of brain homogenate from T201S case 1 demonstrated a PrP^Sc^ type corresponding to type 2 PrP^Sc^ of the London classification seen in patients with sCJD (Hill et al., 2003) (Fig. 2A). Type 2 PrP^Sc^ shows a predominance of monoglycosylated PrP (Hill et al., 2003), which contrasts markedly with the distinctive glycoform ratio of mutant PrP^Sc^ seen in IPD E200K (Fig. 2B) (Hill et al., 2006). These findings indicate that the T201S missense coding change does not impart conformational preferences to PrP^Sc^ in the same way that E200K does (Hill et al., 2006), (Asante et al., 2009).

### 3.6 Effect of T201S on prion protein structure

**Fig. 3:**
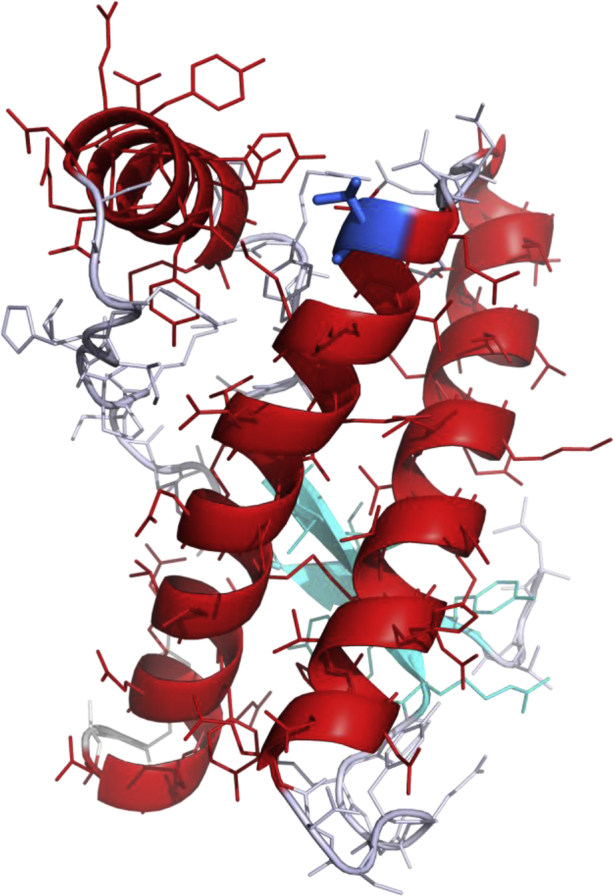
Location of the T201S variant in the structure of human PrP^C^. PrP^C^ is displayed as a “ribbon” representation of its secondary structure, together with side chain groups. α-Helices are colored red and β-strands colored cyan (residues 125–225 are displayed). Residue 201 is located at the start of the third α-helix and is colored in blue with its threonine side chain displayed in stick representation. This figure was prepared using PyMOL (PyMOL Molecular Graphics System, Schrödinger, LLC). Abbreviation: PrP, prion protein. (For interpretation of the references to color in this figure legend, the reader is referred to the Web version of this article.)

The threonine to serine substitution studied here is also conservative, as both these amino acids are uncharged, polar, and of similar size; serine being slightly smaller due to the substitution of a proton for the methyl group found in the threonine side chain. Furthermore, X-ray and nuclear magnetic resonance studies of recombinant PrP^C^(Antonyuk et al., 2009, Biljan et al., 2013) show that T201 is situated at the start of helix 3 of the PrP, with its side chain predominantly solvent exposed rather than within the protein core; thus, unlikely to destabilize PrP^C^ (Fig. 3).

### 3.7 Computational (in silico) predictions

A range of sequence- and structure-based in silico tools is available to assist the interpretation of novel missense variants. It is however recognized that these computational algorithms are inclined to overestimate the damaging effect of missense variants, particularly in the context of variants of milder impact.

Here, Polymorphism Phenotyping version 2 (PolyPhen-2) (Adzhubei et al., 2013) and Sorting Intolerant From Tolerant (SIFT) (Kumar et al., 2009) predicted that the T201S mutation to be *possibly damaging* or *deleterious*, respectively; its Combined Annotation Dependent Depletion (CADD) (Kircher et al., 2014) score of 26 ranks it within 1% of the most deleterious mutations. While these in silico tools are unanimous in their predictions for highly penetrant mutations such as P102L, D178N, and E200K (*probably damaging* by PolyPhen-2, *deleterious* by SIFT, and score >30 by CADD), predictions for other *PRNP* missense variants, both benign and pathogenic, are somewhat mixed. For example, the benign V209M is predicted to be *benign* by PolyPhen-2, *deleterious* by SIFT, and a CADD score of 20.2; the incompletely penetrant V210I is predicted to be *benign* by PolyPhen-2, *tolerated* by SIFT, and a CADD score of 13.53; the highly penetrant A117V is predicted to be *probably damaging* by PolyPhen-2 and CADD score 23.3, but *tolerated* by SIFT. Hence, this illustrates why sequence variants of *PRNP* should not be evaluated solely by in silico tools.

### 3.8 Estimating the penetrance of T201S

T201S was found in a single individual in the Broad Institute's Genome Association Database (gnomAD) (Lek et al., 2016) of 123,125 individuals (1 in 246,250 alleles). By leveraging this large-scale population database, we then used methods for calculating the baseline risk of CJD previously described in Minikel et al. (2016) and computed the upper bound of the 95% confidence interval (CI) using the Wilson Interval (Minikel et al., 2016). The total CJD alleles from sequenced *PRNP* are derived from the sum of alleles in sequenced CJD cases in Minikel et al. (2016) (years 1990–2013) and additional alleles (n = 844) from sequenced CJD cases at the Medical Research Council Prion Unit (years 2014 to present). The estimated penetrance of T201S using this approach is 0.45% (95% CI 0.02%, 9.35%).

## 4 Discussion

The clinical picture of IPD caused by highly penetrant *PRNP* mutations such as E200K, D178N, and P102L can be indistinguishable from that of sCJD. Both our cases had acute clinical onsets within the peak age range of onset reported for sCJD, followed by rapid neurological decline and death within 3 months. Together with restricted diffusion affecting the anterior basal ganglia and cortical ribboning on MRI, generalized periodic complexes on EEG (case 2), cerebrospinal fluid protein 14.3.3 positivity (case 1), and neuropathological findings (case 1), both these patients fulfilled diagnostic criteria for probable sCJD (Zerr et al., 2009) had *PRNP* sequencing not been carried out.

Given that neither case is derived from multiplex families with affected individuals, principles of Mendelian segregation cannot be applied to implicate the T201S mutation in causing familial CJD. In addition, like many rare gene variants, it is not possible to apply practice guidelines such as the American College of Medical Genetics and Genomics for novel gene variants (Richards et al., 2015) in the case of T201S, due to insufficient data to combine criteria for stratification (Table 1). With T201S in particular, there is lack of segregation, functional, de novo, and computational and predictive data to satisfy the stipulated American College of Medical Genetics and Genomics criteria. Moreover, it should be pointed that highly penetrant *PRNP* mutations cause disease by unknown mechanisms that result in a conformational structural change, rather than by simple loss- or gain-of-function mechanisms in which functional and computational data can be more tractable to study in cellular models.

**Table 1:** Lines of evidence used to estimate T201S causality in comparison to ACMG guidelines

Supportive features of variant pathogenicity			
	ACMG guidelines	Pathogenic *PRNP* variants	T201S
Population data	Absent in population databases (NB partially penetrant variants may be rarely detected in large population samples)	Yes	No
	Prevalence in affected patients statistically increased over controls	Yes	Yes, but penetrance is close to zero
Computational data	Multiple lines of computational evidence support a deleterious effect on the gene/gene product	Not always consistent	Yes
	Novel missense change at an amino acid residue where a different pathogenic missense change has been seen before or protein length changing variant	Yes	No
	Same amino acid change as an established variant	Yes	No
	Predicted null variant in a gene where LOF is a known mechanism of disease	Not applicable	Not applicable
Functional data	Missense in gene with low rate of benign missense variants and pathological missenses common	Rare missense variants are common in *PRNP*	Rare missense variants are common in *PRNP*
	Mutational hotspot or well-studied functional domain without benign variation	Not applicable	Not applicable
	Well-established functional studies show a deleterious effect	No simple functional model	No simple functional model
Segregation data	Cosegregation with disease in multiple affected family members	Yes	No
De novo data	De novo with or without paternity and maternity confirmed	Yes in some cases	Unknown
Allelic data	Detected in trans with a pathogenic variant (recessive only)	Not applicable	Not applicable
Other data	Patient's age, phenotype, or family history highly specific for gene	Very young onset (<40) makes sCJD less likely. Sometimes specific IPD phenotypes, for example, fatal insomnia, PrP systemic amyloidosis or Gerstmann-Straussler-Scheinker–associated clinical picture	No
	Filamentous PrP deposition in white matter on autopsy	Often seen	Not seen
	Western blot appearances	Diglycosylated PrP predominates	Monoglycosylated PrP predominates (similar to sCJD)
	Protein structure considerations	Protein structure analysis sometimes predicts destabilization but not consistently.	Protein structure analysis does not predict destabilization.

Key: ACMG, American College of Medical Genetics and Genomics; IPD, inherited prion diseases; LOF, loss of function; NB, nota bene; PrP, prion protein; sCJD, sporadic Creutzfeldt-Jakob disease.

Interrogation of PrP structure and its perturbations by missense mutations have largely focused on stabilization/destabilization of the native PrP structure. However, these studies using recombinant PrP show that stabilization/destabilization of the native PrP^C^ structure is not consistently observed across all known pathogenic mutations (Liemann and Glockshuber, 1999). Furthermore, observations made from these models may not be applicable to real life, as recombinant PrP is unanchored to the cell membrane and unglycosylated, and certainly could fail to capture all of the folding problems encountered in vivo. Alternatively, the disease-associated mutations may primarily affect the stability of more relevant on-pathway folding intermediates (Hart et al., 2009). As such, the pathogenicity of T201S cannot be completely ruled out based solely on PrP structural considerations, despite the seemingly minor perturbation of the native PrP^C^ structure by the T201S substitution.

Nevertheless, we showed that it is possible to produce both qualitative and quantitative estimates of pathogenicity and penetrance for T201S respectively, by harnessing data from multiple lines of evidence specific for prion disease. Different prion strains can propagate in the same host to produce different disease phenotypes and appear to be encoded by distinct abnormal PrP conformations and assembly states (Collinge, 2016, Collinge and Clarke, 2007, Prusiner, 1998). Different human PrP^Sc^ isoforms associated with phenotypically distinct forms of human prion disease (molecular strain types) have considerable diagnostic utility and are classified by both the fragment size and ratio of the 3 principal PrP bands seen after protease digestion (Hill et al., 2006, Hill et al., 2003). Variations in the primary sequence of human PrP profoundly affect the ability of the expressed protein to propagate particular prion strains through conformational selection (Collinge, 1999, Collinge, 2001, Collinge, 2016, Collinge and Clarke, 2007, Wadsworth and Collinge, 2011, Wadsworth et al., 2004, Wadsworth et al., 2010). The codon 129 polymorphism (either methionine or valine) determines the ability of wild-type human PrP to propagate particular prion strains in patients with sporadic or acquired forms of prion disease while highly penetrant missense mutations that cause IPD (Mead, 2006), for example, P102L, E200K, and D178N, impose additional conformational preferences for PrP assemblies, resulting in PrP^Sc^ molecular strain types that are distinct from those propagated in patients with sporadic or acquired etiologies (Asante et al., 2015, Asante et al., 2009, Hill et al., 2006, Wadsworth et al., 2010, Wadsworth et al., 2006). Immunoblot and glycoform analyses showed that PrP^Sc^ from T201S brain tissue resembled that of type 2 sCJD 129MM rather than that seen in highly penetrant *PRNP* point mutations, for example, E200K. This dissimilarity is further reinforced by the absence of white matter filamentous PrP deposits on neuropathology, although it can be argued that this observation has limited negative predictive value for variants toward the carboxy-terminal of *PRNP* (Reiniger et al., 2013). PrP structural analyses also suggest that the resulting amino acid substitution is not expected to impart a significant change in PrP conformation.

Finally, we argue that the estimated penetrance of 0.45% (95% CI 0.02%, 9.35%) calculated by leveraging the gnomAD (Lek et al., 2016) population data indicate that T201S is at most a low-risk gene variant for CJD. If we arbitrarily consider a central estimate of 10% or higher as the clinically significant threshold at which to refer for predictive testing, an excess of 44 T201S alleles in the CJD-diseased population would need to be observed, provided that the other variables remain constant. It is highly unlikely that new variants discovered in routine disease surveillance will achieve these counts in the foreseeable future.

One important factor that determines the accuracy of penetrance estimation is ascertainment of the true population allele count; not only can this be imprecise for singletons of extremely low frequency (such as T201S) but also biased toward underestimation. Within the Exome Aggregation Consortium and gnomAD data set, this is exemplified by the shift in calculated penetrance from 0.22% (95% CI 0.01%, 4.56%) to 0.45% (95% CI 0.02%, 9.35%), when the original Exome Aggregation Consortium database expanded into the gnomAD in which the allele count doubled from 121,384 to 246,250 alleles. Even more strikingly, it was pointed out that 69% of very rare singletons for Europeans (6503 exomes) in the Exome Sequencing Project were not identified again in the Exome Aggregation Consortium database, despite a 10-fold expansion (Lek et al., 2016). Hypothetically, if this holds true for gnomAD, the true allele frequency of a rare singleton such as T201S could be 1 in 2.5 million or lower, raising the upper limit of the 95% CI to 94% (or higher) and rendering the estimation meaningless. Bearing this in mind, the mere presence of T201S in large gnomAD database does not absolve T201S as a highly penetrant variant. However, the late ages at onset, lack of family history, lack neuropathological features of IPD, and molecular strain typing (glycoform ratio) reminiscent of sCJD are all in line with the estimation that T201S is either benign or at most a low-risk variant below “clinically significant” threshold.

## 5 Conclusion

At present, although we cannot conclusively determine whether T201S is a nonpathogenic variant co-occurring with sCJD or a low-risk non-Mendelian variant, its estimated penetrance is insufficient to justify routine predictive *PRNP* testing in individuals at risk of T201S. The results of our analyses were discussed with the family of case 2, and the above conclusion was conveyed. Nevertheless, her offspring requested a referral to a clinical geneticist, who arrived at the same conclusion, and similarly advised against predictive *PRNP* testing. We recognize that further research, particularly expanding the coverage of molecular strain typing to include other *PRNP* sequence variants of varying pathogenicity and penetrance, is required to refine it as a discriminating tool. In addition, future expansion of large-scale population genomic databases in tandem with assiduous surveillance and sequencing of *PRNP* in CJD cases will further hone the precision of estimating true penetrance of rare sequence variants.

## Disclosure statement

JC is a Director and shareholder and JDFW is a shareholder of D-Gen Limited, an academic spinout company working in the field of prion disease diagnosis, decontamination, and therapeutics. D-Gen supplied the ICSM 35 antibody that was used in this study.
