# Supplementary material for: Evaluating the causality of novel sequence variants in the prion protein gene by example
Source: Neurobiol Aging. 2018 Nov;71:265.e1–7. doi: 10.1016/j.neurobiolaging.2018.05.011 (PMC6175539; doi:10.1016/j.neurobiolaging.2018.05.011)
Supplement: Supplementary Table S1 [file mmc1.doc]

| **Variant** | **Number** |
| --- | --- |
| 1-OPRD | 7 |
| 4-OPRI | 2 |
| 5-OPRI | 2 |
| 6-OPRI | 3 |
| 8-OPRI | 1 |
| G54S | 2 |
| P102L | 5 |
| A117A | 27 |
| A117V | 2 |
| N171S | 1 |
| D178N | 3 |
| E196K | 1 |
| E200K | 14 |
| D202D | 1 |
| V210I | 1 |
| E219K | 1 |

**Table S1 Allele spectrum of additional sequenced cases (total = 844 alleles) at the MRC Prion Unit from January 2014 to April 2018**
